# Progression of stroke risk in patients aged <65 years diagnosed with atrial fibrillation: a cohort study in general practice

**DOI:** 10.3399/BJGP.2022.0568

**Published:** 2023-07-25

**Authors:** Silvia C Mendonça, Duncan A Edwards, Jenny Lund, Catherine L Saunders, Jonathan Mant

**Affiliations:** Wellcome Trust clinical PhD fellow in primary care;; Wellcome Trust clinical PhD fellow in primary care;; Wellcome Trust clinical PhD fellow in primary care;; Primary Care Unit, Department of Public Health and Primary Care, University of Cambridge, Cambridge.; Primary Care Unit, Department of Public Health and Primary Care, University of Cambridge, Cambridge.

**Keywords:** anticoagulation, atrial fibrillation, primary care, risk factors, stroke risk

## Abstract

**Background:**

As a result of new technologies, atrial fibrillation (AF) is more likely to be diagnosed in people aged <65 years.

**Aim:**

To investigate the risk of someone diagnosed with AF aged <65 years developing an indication for anticoagulation before they reach 65 years.

**Design and setting:**

Population-based cohort study of patients from English practices using the Clinical Practice Research Datalink, a primary care database of electronic medical records.

**Method:**

The study included patients aged <65 years newly diagnosed with AF. The CHA_2_DS_2_-VASc score was derived at time of diagnosis based on patients’ medical records. Patients not eligible for anticoagulation were followed up until they became eligible or turned 65 years old. The primary outcome of interest was development of a risk factor for stroke in AF.

**Results:**

Among 18 178 patients aged <65 years diagnosed with AF, 9188 (50.5%) were eligible for anticoagulation at the time of diagnosis. Among the 8990 patients not eligible for anticoagulation, 1688 (18.8%) developed a risk factor during follow-up before reaching 65 years of age or leaving the cohort for other reasons, at a rate of 6.1 per 100 patient-years. Hypertension and heart failure were the most common risk factors to occur, with rates of 2.65 (95% CI = 2.47 to 2.84) and 1.58 (95% CI = 1.45 to 1.72) per 100 patient-years, respectively. The rate of new diabetes was 0.95 (95% CI = 0.85 to 1.06) per 100 patient-years.

**Conclusion:**

People aged <65 years with AF are at higher risk of developing hypertension, heart failure, and diabetes than the general population, so may warrant regular review to identify new occurrence of such risk factors.

## INTRODUCTION

Atrial fibrillation (AF) is a rhythm disturbance of the heart that becomes increasingly common as people age.^1^ AF is a strong risk factor for stroke,^2^ but this risk can be substantially reduced by treatment with anticoagulation.^3^ Recognising that such treatment is not without hazard (of bleeding), National Institute for Health and Care Excellence (NICE) guidelines recommend that treatment with anticoagulation is offered on the basis of risk of stroke.^4^ A widely used score to assess the risk of stroke in patients with AF is the CHA_2_DS_2_-VASc score.^5^

NICE guidelines recommend that anticoagulation should be considered or offered to all patients with AF except those <65 years with no risk factors other than their sex (women with AF are at higher risk of stroke than men with AF, but simply being female doesn’t warrant/require anticoagulant treatment).^4^ This raises the question as to how frequently patients who fall into this category (<65 years with AF and no risk factors) should be reviewed so that anticoagulation can be considered should their risks change.

In the UK, the Quality and Outcomes Framework encourages annual review of such patients,^6^ but there is little evidence to inform this aspect of clinical practice. A cohort study in Denmark of people with AF aged 30–65 years confirmed the utility of the CHA_2_DS_2_-VASc score in this population, and that all the individual risk factors remained independent predictors of stroke in this age group.^7^ With regard to the risk of development of the individual CHA_2_DS_2_-VASc risk factors in people with AF, a systematic review of cohort studies found that AF is associated with increased future risk of heart failure and peripheral vascular disease.^8^ For hypertension and diabetes mellitus, the focus has been on the risk of AF in association with these conditions, rather than the other way round.^9^^,^^10^ Thus, there is only limited evidence on the risk of development of risk factors for stroke in people who have AF. This is likely to be a growing issue in the future as a result of the increased use of wearable devices that can incidentally detect AF.^11^^,^^12^ Therefore, the aim of this study was to quantify the proportion of people <65 years in general practice newly diagnosed with AF who had an indication for anticoagulation, and, for those who did not, to quantify their risk of developing an indication for anticoagulation before their 65th birthday, and the risk of development of the individual CHA_2_DS_2_-VASc risk factors.

## METHOD

### Data source

The Clinical Practice Research Datalink (CPRD) GOLD is a database of electronic primary care records in the UK, based on patients attending GP practices, which use the Vision computer system. The CPRD has been shown to be a nationally representative sample corresponding to about 7% of the UK population in 2013.^13^ The data include coded information on medical diagnoses, referrals, tests, and all prescriptions issued at the practice.

**Table:** How this fits in

New technologies are likely to result in younger people being diagnosed with atrial fibrillation who do not require anticoagulation treatment at diagnosis. There are few data to inform follow-up of such people. This study found that the risk of developing hypertension and heart failure (indications for anticoagulation) was high in this group, suggesting that more frequent review of patients aged <65 years with atrial fibrillation is required compared with the general population.

### Study population

This study used a cohort included in a previous study.^1^ The cohort included patients from English practices newly diagnosed with AF between 1 January 2004 and 31 December 2018, identified using diagnostic codes for AF. The current study only considered patients who were <65 years at the time of AF diagnosis.

### CHA_2_DS_2_-VASc risk score

The CHA_2_DS_2_-VASc score was used to determine patients’ eligibility for anticoagulation. The risk score was calculated directly for each patient, rather than relying on the coding of the CHA_2_DS_2_-VASc itself in the patient record, again based on diagnostic codes in clinical records for each condition. The code lists for these diagnoses were developed as part of a project on the epidemiology of multimorbidity (see Supplementary Table S1 for details).^14^^,^^15^ For hypertension, diagnostic codes were used rather than blood pressure level.

### Statistical analysis

The proportion of patients with AF <65 years who are eligible for anticoagulation at the time of diagnosis was calculated based on current NICE guidelines that anticoagulation should not be offered to people in this age group with AF and no risk factors other than their sex (that is, a CHA_2_DS_2_-VASc score of 0 for males or 1 for females).^4^

For those who were ineligible for anticoagulation at diagnosis, the rate at which they become eligible was calculated. The start of follow-up was the date of AF diagnosis and the end of follow-up was the earliest of the last day the patient was registered at the practice, date of death, the last day the practice contributed to the CPRD, 31 December 2018 (the end of study date), or the date the patient turned 65 years old. The primary outcome was development of the first CHA_2_DS_2_-VASc risk factor.

As secondary outcomes, the risk of each risk factor occurring separately (heart failure, hypertension, diabetes, stroke/transient ischaemic attack/systemic embolism, and vascular disease) was examined, independently of another risk factor having already occurred during follow-up. In this analysis of secondary outcomes all conditions developed during follow-up were included instead of just the first one, and individual time at risk takes into account when each risk factor occurred.

Incidence rates were calculated as the number of patients who develop relevant conditions during follow-up (numerator) divided by the total person-years at risk (denominator). The numerator includes only patients who develop relevant conditions during follow-up and excludes those that become eligible when turning 65 years old. The denominator is the sum of the person-years at risk from all eligible patients. Stratified incidence rates by age group were calculated through the use of a Lexis expansion where each person time at risk was split according to their age group.^16^ Ninety-five per cent confidence intervals (95% CI) were reported using the Poisson distribution.

As a post-hoc analysis, the evolution of risk factors over the first 10 years after AF diagnosis were plotted in a Kaplan‒Meier graph, which shows the estimated probability of having no risk factors (estimated survival function) for the primary and secondary outcomes.

## RESULTS

**Figure 1.**
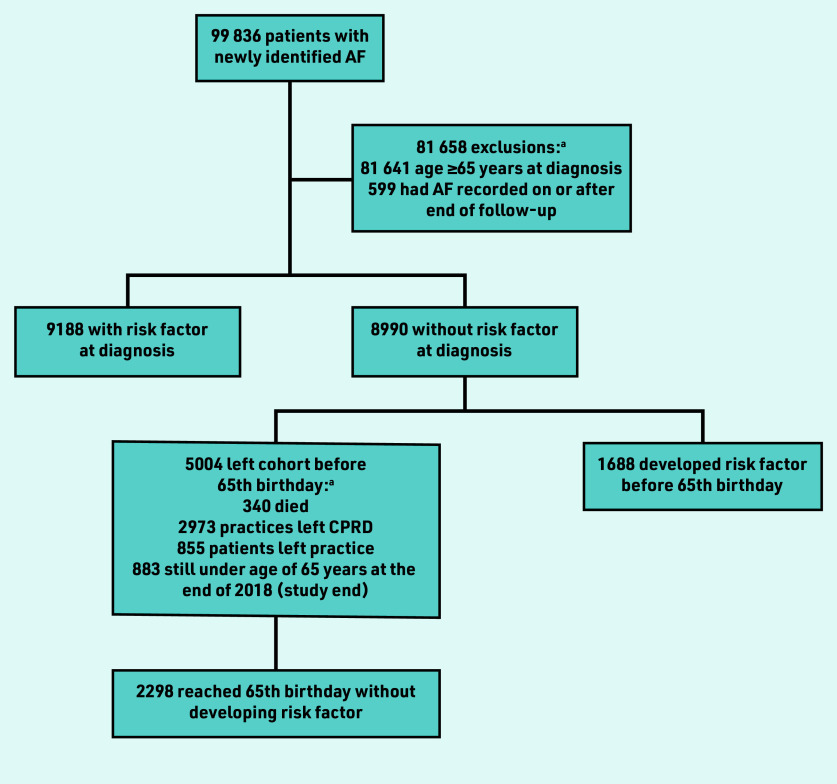
*Flowchart showing the number of patients included for each analysis aim, starting from the number of patients included in a previous study.^1^* *^a^There is overlap in the categories specified so they do not sum to 5004 (that is, a few patients were double counted here, for example, if the patient record included a date of death and a date the patient left the practice, and these were the same, the authors did not give precedence to one or the other). AF = atrial fibrillation. CPRD = Clinical Practice Research Datalink.*

During the time period 2004–2018, 18 178 new cases of AF were identified in people <65 years (18.2% of all new cases) (Figure 1).^1^ A little over half of these patients (50.5%, *n* = 9188) had a pre-existing risk factor for AF, and so were immediately eligible for anticoagulation.

The presence of at least one risk factor was more likely in older patients, with 60.7% of people aged 60–64 years (*n* = 4698) having a risk factor, compared with 29.1% of people aged 40–49 years (*n* = 792) (Table 1). Hypertension was the most common risk factor (present in 38.8%, *n* = 7062), and heart failure the least common (6.09%, *n* = 1107).

**Table 1.** Presence of risk factors for stroke by age and sex in 18 178 participants aged <65 years at time of diagnosis

	**No risk factors**		**At least one risk factor**		**Risk factors present at diagnosis**									

					**Hypertension**		**Diabetes mellitus**		**Vascular disease**		**Stroke/TIA**		**Congestive heart failure**	

**Patient group**	***n***	**%**	***n***	**%**	***n***	**%**	***n***	**%**	***n***	**%**	***n***	**%**	***n***	**%**
All patients	8990	49.5	9188	50.5	7062	38.8	2155	11.9	1425	7.84	1231	6.77	1107	6.09

Age group														
40–49	1928	70.9	792	29.1	570	3.14	173	0.95	87	0.48	93	0.51	106	0.58
50–59	4014	52.0	3698	48.0	2821	15.52	919	5.06	532	2.93	442	2.43	455	2.50
60–64	3048	39.3	4698	60.7	3671	20.19	1063	5.85	806	4.43	696	3.83	546	3.00

Female														
40–49	532	75.1	176	24.9	129	0.71	40	0.22	13	0.07	22	0.12	26	0.14
50–59	1293	55.6	1034	44.4	818	4.50	230	1.27	96	0.53	149	0.82	109	0.60
60–64	1066	41.7	1490	58.3	1234	6.79	299	1.64	144	0.79	221	1.22	129	0.71

Male														
40–49	1396	69.4	616	30.6	441	2.43	133	0.73	74	0.41	71	0.39	80	0.44
50–59	2721	50.5	2664	49.5	2003	11.02	689	3.79	436	2.40	293	1.61	346	1.90
60–64	1982	38.2	3208	61.8	2437	13.41	764	4.20	662	3.64	475	2.61	417	2.29

*TIA = transient ischaemic attack.*

**Figure 2.**
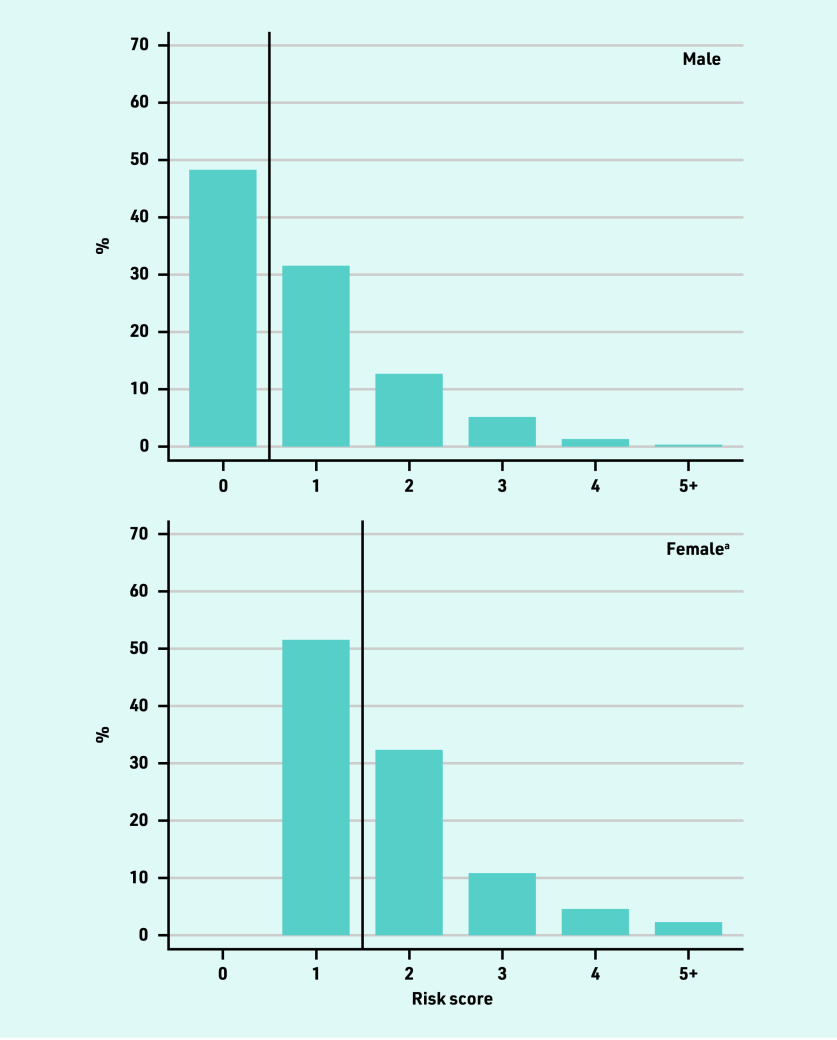
*Distribution of CHA_2_DS_2_-VASc risk score at time of atrial fibrillation diagnosis in patients <65 years, stratified by sex.* *^a^1 point is assigned in CHA2DS2-VASc for being female, hence no females have a risk score of 0.*

The prevalence of all risk factors rose with age. In each age stratum, a higher proportion of males than females had a risk factor, but this difference was not substantial. The distribution of total CHA_2_DS_2_-VASc score was similar in males and females, with the female score shifted to the right by one — a feature of the assignment of a score of 1 given for female sex (Figure 2, and Supplementary Figure S1).

Most of the 8990 participants without an indication for anticoagulation were males (67.8%, *n* = 6099), reflecting their higher prevalence of AF (Table 1). Over the period of follow-up, 1688 (18.8%) participants developed a risk factor at a rate of 6.13 (95% CI = 5.85 to 6.43) per 100 patient-years before reaching the age of 65 years or leaving the cohort for other reasons (Table 2). The rate was lowest (4.75, 95% CI = 4.18 to 5.41 per 100 patient-years) in people aged 40–49 years, and highest (7.10, 95% CI = 6.60 to 7.64 per 100 patient-years) in people aged 60 to 64 years. Males developed a risk factor at a higher rate than females (6.75 per 100 patient-years versus 4.85 per 100 patient-years).

**Table 2.** Risk of becoming eligible for anticoagulation in patients in the subsample (*n* = 8990) diagnosed with atrial fibrillation <65 years who were low risk at diagnosis

**Patient group**	**Total person-years**	**Number (%) of patients who become eligible for anticoagulation during follow-up**	**Rate of becoming eligible for anticoagulation during follow-up per 100 patient-years (95% CI)**
All patients	27 524	1688 (18.8)	6.13 (5.85 to 6.43)

Male	18 522	1251 (20.5)	6.75 (6.39 to 7.14)

Female	9002	437 (15.1)	4.85 (4.42 to 5.33)

Age group^a^			
40–49	4796	228 (11.8)	4.75 (4.18 to 5.41)
50–59	12 718	749 (18.7)	5.89 (5.48 to 6.33)
60–64	10 011	711 (23.3)	7.10 (6.60 to 7.64)

a *Age group in which the risk factor was diagnosed.*

**Figure 3.**
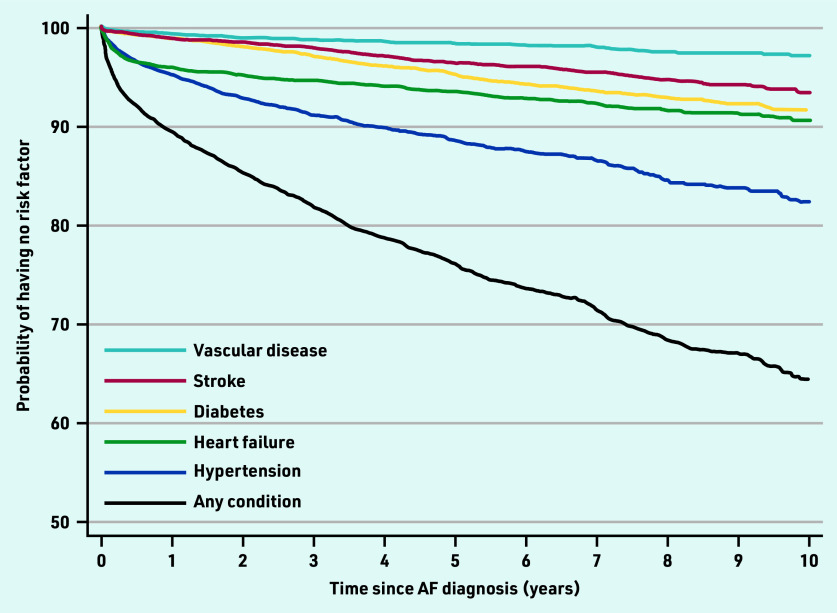
*Kaplan–Meier graph showing the evolution of risk factors over the first 10 years after diagnosis of atrial fibrillation (see Supplementary Table S2 for details of summary statistics used in this graph). AF = atrial fibrillation.*

Hypertension was the most common risk factor to develop in the study cohort without an initial indication for anticoagulation, with an incidence of 2.65 (95% CI = 2.47 to 2.84) per 100 patient-years, followed by heart failure (1.58 per 100 person-years, 95% CI = 1.45 to 1.72), and diabetes (0.95 per 100 person-years, 95% CI = 0.85 to 1.06). The incidence of stroke and vascular disease was 0.71 per 100 person-years (95% CI = 0.62 to 0.81) and 0.34 per 100 person-years (95% CI = 0.29 to 0.41), respectively. Development of these risk factors over time is shown in Figure 3 (see also Supplementary Table S2 for details). The risks of a new risk factor being identified are particularly high in the first year following the diagnosis of AF. This is driven largely by new diagnoses of hypertension and heart failure.

## DISCUSSION

### Summary

This study found that half of people <65 years diagnosed with AF have an indication for anticoagulation. The remaining half become eligible for anticoagulation at a rate of 6% per annum, most commonly as a result of developing hypertension or heart failure.

### Strengths and limitations

To the authors’ knowledge, this is the first study to look at the risk of developing risk factors for stroke in people with AF. The study included just under 9000 participants who did not have a risk factor at diagnosis, so risks could be reported with reasonable precision in terms of width of confidence intervals.

This cohort, while representative of patients in UK primary care, will have had their AF largely diagnosed through traditional methods (namely, using a 12-lead electrocardiogram [ECG]) because wearable devices such as smart watches and fitness trackers did not have the AF detection function enabled outside trials in the UK by 2018, and NICE only recognised the use of other ECG technology to diagnose paroxysmal AF in 2021.^4^ This group may not be representative of people with AF in the future, who will increasingly be diagnosed with novel technologies.

Validity of the current analysis depends on the accuracy of GP coding of both AF and the risk factors for AF, which is prone to error.^17^^,^^18^ For example, if blood pressure had been used in addition to hypertension codes, it would have increased the sensitivity to hypertension, but with a loss of specificity. It is possible that the association between the diagnosis of AF and development of heart failure and hypertension in the first months following diagnosis reflects reverse causality. In this analysis, it is not possible to distinguish to what extent the risks of development of risk factors for stroke relate to the AF, or to other factors such as obesity and socioeconomic deprivation, which are associated both with AF and risk factors for stroke.^19^^,^^20^

Over half of the participants left the cohort before reaching the age of 65 years. While this was mostly as a result of their practice leaving the CPRD, it is plausible that this might have affected the observed rates of risk factor occurrence, if practices that left the CPRD were systematically different from practices that stayed in it. Since detection of risk factor occurrence depends on GP diagnosis, the true risks are likely to be higher.

### Comparison with existing literature

The low risk of stroke in this study is below that observed for people with a CHA_2_DS_2_-VASc score of 0 or 1 in a Danish cohort,^5^ thus confirming that it is not appropriate to offer anticoagulation to such people.

It is difficult to estimate what would be the incidence rates of the risk factors in people not with AF, due to a lack of studies of incidence of these risk factors in equivalent populations over a similar time period. Nevertheless, a high incidence of heart failure was observed compared with those not in AF in this age group,^21^ consistent with the five-fold increase in the risk of heart failure in AF found in observational studies.^8^ The observed incidence of diabetes in this study was about twice what would be expected in a similar aged population without AF.^22^ Similarly, the incidence of hypertension was approximately double the rate of new cases of hypertension diagnosed through health checks.^23^

### Implications for research and practice

While NICE guidance gives recommendations for the symptomatic management of AF in people <65 years,^4^ there is only limited evidence as to how to follow up stroke risk in people diagnosed with AF who do not meet the threshold for anticoagulation treatment.

This cohort study of just under 9000 of such people was conducted to inform this issue. The risk of developing heart failure, hypertension, or diabetes in this population seems higher than would be expected in the non-AF population, particularly in the case of heart failure. This suggests that standard care should include regular assessment for these risk factors so that they can be managed, and anticoagulation to prevent stroke can be started promptly. This is consistent with the current Quality and Outcomes Framework indicator that rewards re-assessing stroke risk annually for people with AF whose CHA_2_DS_2_-VASc score is less than 2, although it does not prompt active case finding of any of the CHA_2_DS_2_-VASc risk factors.^6^ More formal epidemiological studies of the association of AF with these risk factors would enable firmer recommendations to be made.

## Data

The study uses data from the Clinical Practice Research Datalink (CPRD). The CPRD does not allow the sharing of patient-level data. The code lists used in this study are part of a set of lists made available at https://www.phpc.cam.ac.uk/pcu/research/research-groups/crmh/cprd_cam/codelists/.
